# Victimization among adolescents of female sex workers: findings from the children of at-risk parents (CARP) study in Uganda

**DOI:** 10.1186/s12887-023-04131-w

**Published:** 2023-06-20

**Authors:** Simple Ouma, Catherine Abbo, Nakita Natala, Molly McCoy, Maria Kroupina

**Affiliations:** 1 Department of Psychiatry, School of Medicine, Makerere University College of Health Sciences, Kampala, Uganda; 2 Department of Research, The AIDS Support Organization (TASO), Kampala, Uganda; 3 Department of Psychiatry and Behavioral Sciences, University of Minnesota, Minnesota, USA; 4 Department of Pediatrics, University of Minnesota, Minnesota, USA

**Keywords:** Adolescents, Female sex workers, Children of at-risk parents, Lifetime victimization

## Abstract

**Background:**

Female sex workers (FSWs) live and work in high-risk environments, experience high levels of adversity, and have multigenerational trauma that can negatively affect their children. Yet not much is known about the prevalence of victimization (i.e., exposure to maltreatment and trauma) among children of FSWs. This study compared the prevalence of lifetime victimization among adolescents of FSWs and adolescents of non-FSWs in Gulu City, Northern Uganda.

**Methods:**

A comparative cross-sectional study was conducted among adolescents (10–17 years) enrolled in the Children of At-Risk Parents (CARP) study. This study included 147 adolescents of FSWs and 147 adolescents of non-FSWs selected for comparison in Gulu City, Northern Uganda. The adolescents of FSWs were identified through their mothers using respondent-driven sampling. Data on the residence of FSWs guided a proportionate stratified sampling of adolescents of non-FSWs. Using the Juvenile Victimization Questionnaire, we screened for 34 different types of victimization during participants’ lifetimes. Percentage point differences within groups of adolescents and comparison between adolescents of FSWs and non-FSWs were calculated using STATA version 14.1. Statistical significance was set to p < 0.05.

**Results:**

99.3% of the participants experienced at least one form of lifetime victimization. The median number of lifetime victimizations was 12.4. Overall, lifetime victimization was higher among adolescents of FSWs than non-FSWs (13.4 vs. 11.5), male vs. female adolescents (13.4 vs. 11.9), and older [14–17 years] vs. younger (10–13 years) adolescents (14.0 vs. 11.7). Further, more adolescents of FSWs experienced lifetime victimization in the following domains and subdomains, all of which were statistically significant: kidnap (15.8% vs. 4.8%), emotional abuse (65.8% vs. 50.0%), emotional neglect (37.4% vs. 21.1%), physical intimidation (10.2% vs. 4.1%), relational aggression (36.4% vs. 18.4%), verbal aggression (68.7% vs. 46.9%), sexual victimization (31.3% vs. 17.7%), verbal sexual harassment (20.4% vs. 5.4%), exposure to murder scene (42.9% vs. 26.5%), witness to domestic violence (39.5% vs. 26.5%), and witness to the murder of relatives (31.3% vs. 21.1%). Conversely, more adolescents of non-FSWs experienced caregiver victimization than the adolescents of FSWs (98.0 vs. 92.5; p < 0.05).

**Conclusions:**

Childhood victimization is highly prevalent in Northern Uganda and disproportionately affects the adolescents of FSWs. Therefore, government and development partners should urgently develop policies and interventions targeting prevention, early detection, and timely management of victimization in this vulnerable population.

**Supplementary Information:**

The online version contains supplementary material available at 10.1186/s12887-023-04131-w.

## Background

Childhood victimization is a public health crisis that affects people at all levels and can lead to lasting health and social problems [1, 2]. Victimization encompasses adverse events like physical and emotional abuse and neglect, sexual abuse, bullying, property violence, community violence, peer/sibling violence, and witnessed/indirect violence [3, 4]. Globally, 70% of the population experienced at least one victimization during their lifetime [5]. Victimization mostly occurs during childhood, with 50% of children (2–17 years) experiencing at least one form of victimization each year [6]. A recent systematic review noted that the highest rate of child maltreatment was in Africa yet only a small proportion of studies on child maltreatment are from the continent [7], thus revealing the need for an urgent investment of resources to study, prevent, and manage this vice in Africa. In Uganda, a previous report indicated that three-quarters (75.4%) of adolescents experience at least one form of childhood victimization [8]. The situation could be more pronounced among adolescents living in conflict-affected Northern Uganda, the site of this study. The extreme poverty and armed conflict in Northern Uganda might have put the Children of At-Risk Parents (CARP) like female sex workers (FSWs) at greater risk of victimization [9–11]. Moreover, FSWs generally work and live in high-risk environments and face extreme levels of adversity and multigenerational trauma [12–14]. In Uganda, sex work is illegal and is not recognized as a form of employment [15, 16], thus exposing the approximately 200 000 FSWs in the country to sexually transmitted infections including HIV, gender-based violence, depression, and poverty [12, 17–19]. The illegality of sex work and the negative life events experienced by the FSWs are perfect precursors for high-level victimization among their children.

Victimization can lead to several short- and long-term impacts on the lives of the affected individuals. In the short term, it can impede a child’s growth and development, distort stress regulation and impair cognition [20–22]. While in the long term, it can lead to physical and mental health problems like substance abuse, obesity, sexually transmitted infections [23], acute stress disorders, posttraumatic stress disorders, mood disorders, anxiety disorders, and personality disorders [19–21]. Likewise, victimization can also exacerbate pre-existing mental health disorders or even precipitate new onsets of mental disorders [24]. If left untreated, it can lead to lasting health problems like intimate partner violence, sexual assault, re-victimization [25], dating violence [26], depression or suicide [27, 28]. Despite the high levels of vulnerability among children of FSWs, there is a paucity of information about their healthcare needs [29], leaving a significant gap in knowledge on their health [30], especially in conflict-affected settings like Northern Uganda [31]. Thus, there is an urgency to understand the healthcare needs of CARP like the FSWs, especially in settings like Uganda where sex work is illegal and culturally despised [15]. Findings will help raise public awareness, inform policies and programs, and support the development of interventions to protect the rights and healthcare needs of children of FSWs. This study aimed to determine the impacts of maternal sex work on adolescent victimization in Northern Uganda.

## Methods

### The aim, design and setting of the study

A comparative cross-sectional study was conducted among 294 adolescents (10–17 years) enrolled in the CARP study comprising 147 adolescents of FSWs and 147 comparative adolescents of non-FSWs in Gulu City, Northern Uganda. Most FSWs in Northern Uganda live and work in urban settings [32], joined sex work due to poverty (89.3%), and operate as mobile sex workers [12] who might have less time for parenting [33]. We collected quantitative data among adolescents of FSWs and adolescents of non-FSWs from the same neighbourhood between November and December 2021.

### Sample size estimation and sampling

The sample size was calculated using formula [34] for comparative proportion (n = 4[Zα_1/2_ + Z_β_]^2^ P(1-P)] / [{P_1_-P_2_}^2^]). Where Zα_1/2_ = 1.96 at type 1 error of 5%, Z_β_ = 0.842 at 80% power, P_1_-P_2_ = difference in the proportion of events between groups, and P = pooled prevalence. Based on the literature, 75.4% of Ugandan adolescents experience victimization [8], assuming a higher level (90%) of victimization among adolescents of FSWs, for equal samples, the sample size per group was 105. Assuming a design effect of 1.25 and adjusting for non-response by 10%, the adjusted sample size was 146 adolescents per group.

We used respondent-driven sampling to reach FSWs with at least one adolescent aged 10–17 years [35, 36]. Respondent-driven sampling is an efficient method for selecting hidden populations in a short period while minimizing costs and maximizing security for both staff and respondents [37]. Due to the complexity and cost associated with reaching adolescents of FSWs through their mothers, each FSW asked to recruit their peers was made are of the eligibility criteria to ensure that they bring only the eligible adolescent-mother pairs for interviews. Initially, we gave out three coupons to three peers of FSWs to recruit nine seeds from nine communities where FSWs were commonly residing. We added two more coupons to the same three peers to recruit six more seeds, each from locations where FSWs commonly solicited sex (brothels, lodges, bars, clubs, streets, and homes). Then, each seed received three coupons to recruit three peers from their social network, and the cycle continued until the desired number of participants was reached. Each recruited FSW came with her oldest eligible adolescent and provided verbal informed consent for her participation and that of the adolescent. Each adolescent assented to participate. We manually monitored coupons through a coupon log notebook.

After collecting data among the adolescents of FSWs, they grouped them by their villages. Thereafter, we utilized a proportionate stratified sampling [38, 39] to reach adolescents of non-FSWs from the same villages as the adolescents of FSWs. From each village, we selected a household of FSWs to act as the starting point for sampling the adolescents of non-FSWs. Initially, we chose the immediate households to the North of the FSWs’ households as the starting points, screened for eligibility, and invited only eligible mother-adolescent pairs for interviews. Subsequently, we selected every fifth household within each village until we reached the required proportions of participants per village. Maternal sex work status was ascertained using three questions as follows: (1) Have you ever received money or goods in exchange for sexual services? (2) If yes, did you receive money or goods in exchange for sexual services in the last year? (3) If yes, do you consider your receipt of money or goods for sexual services as income-generating? Mothers who answered “yes” to all three questions were considered FSWs. Conversely, mothers who answered “no” to question 3 were considered non-FSWs and participated if they lived in the same neighbourhood as the FSWs for a least one year before data collection.

### Data collection and management

A trained senior psychiatric clinical officer and a data clerk collected de-identified data through clinician-administered face-to-face interviews using digitally created case report forms in REDCap electronic data capture tools hosted at the University of Minnesota [40, 41]. REDCap (Research Electronic Data Capture) is a secure, web-based software platform designed to support data capture for research studies, providing (1) an intuitive interface for validated data capture; (2) audit trails for tracking data manipulation and export procedures; (3) automated export procedures for seamless data downloads to common statistical packages, and (4) procedures for data integration and interoperability with external sources. As a backup, we printed paper-based case report forms in the unlikely event of REDCap system failure. Data tools were developed in English, translated into the local language (Acholi) by a language expert, and reviewed for accuracy, cultural adaptation, and language appropriateness. The joint reviewers included the language expert, two Acholi psychiatric clinical officers, an Acholi psychiatric nursing officer, and the principal investigator- a physician from Acholi. Before data collection, tools were pre-tested among ten adolescent-mother pairs. We interviewed both adolescents and mothers since certain family-level factors are risk factors for victimization. We obtained sociodemographic and health characteristics and screened for lifetime victimization using the Juvenile Victimization Questionnaire (JVQ) (4). Though infrequent, JVQ has been used in African settings [42–44]. The basic JVQ contains questions on 34 forms of victimization with yes/no responses and covers five general areas: conventional crime, maltreatment, peer and sibling victimization, sexual victimization, and witnessing and indirect victimization (4).

### Statistical analysis

Characteristics of participants and their mothers were summarized using proportions for categorical variables and mean with standard deviation (SD) for continuous variables with normal distributions or median with interquartile range (IQR) for continuous variables with skewed distributions. To stratify lifetime victimization by age, we grouped participants into younger (10–13 years) or older (14–17 years) adolescents. We examined the differences in victimization between the adolescents of FSWs and their comparators using the chi-square test or Fisher’s exact test when any cell in the two-by-two table had an expected count of less than 5. We tested for the difference in mean using two-tailed t-tests. Associations with p < 0.05 were considered statistically significant. We used STATA version 14.1 for analysis.

### Participant and public involvement

During the dissemination of findings from our previous research among FSWs in the same region [12, 18, 45], FSWs themselves suggested that future studies should look into the mental health of their children. Likewise, we engaged peers of FSWs in identifying the current research topic, as well as planning and conducting the study. We heavily depended on FSWs to recruit eligible fellow FSWs with eligible adolescents. We shall involve FSWs and relevant stakeholders during the dissemination of findings.

## Results

### Socio-demographic characteristics of adolescents in the CARP study

**Fig. 1:**
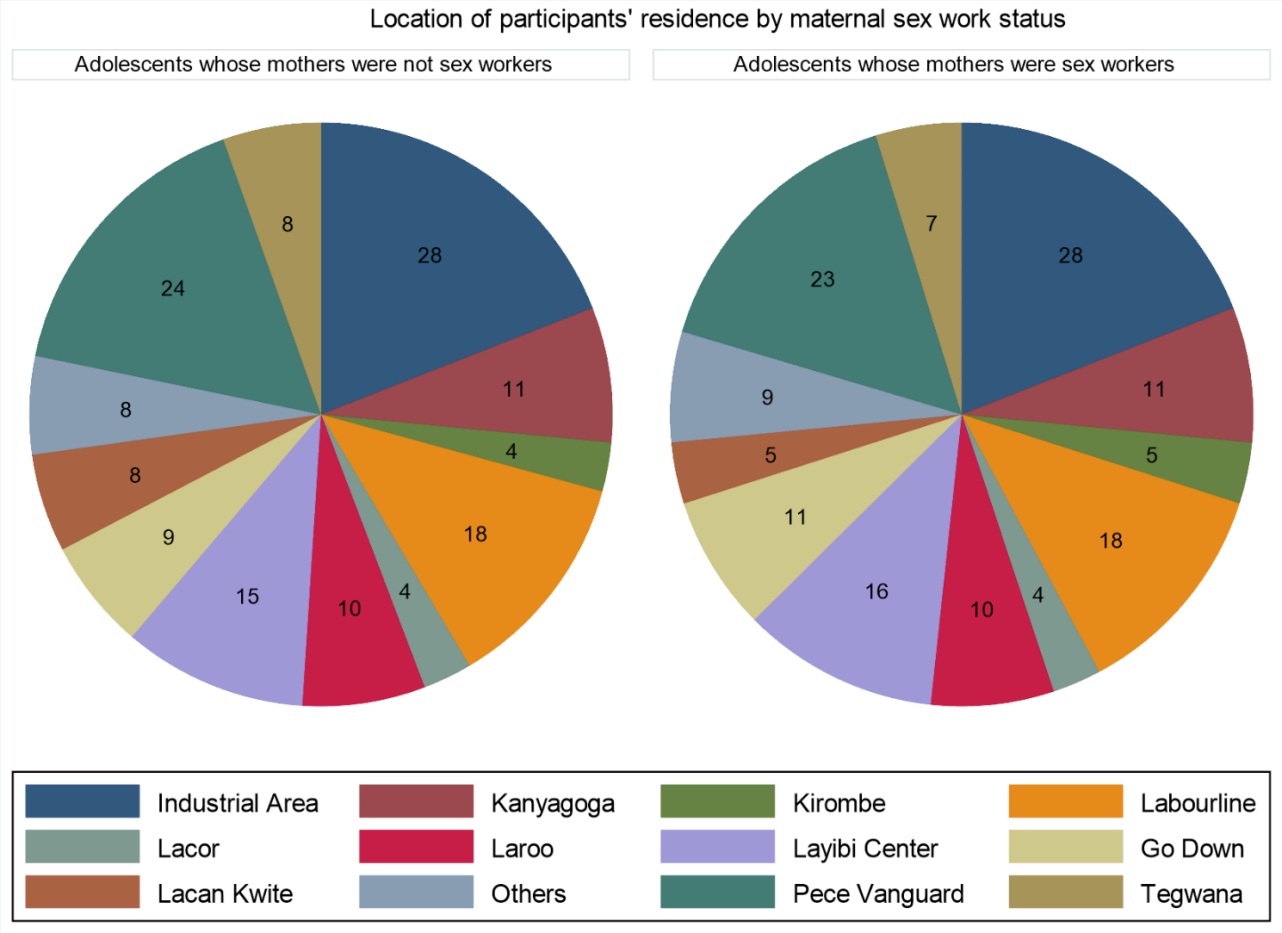
Showing proportionate distribution of participants by residence

A total of 294 adolescents (147 adolescents of FSWs and 147 adolescents of non-FSWs) were selected from the same villages [Fig. 1]) and interviewed. 166 eligible FSWs were contacted to come along with their adolescents but 11.4% (19/166) did not turn up for interviews. Similarly, 171 households were screened to select adolescents of non-FSWs, but 11.1% (19/171) were considered ineligible [five did not have anyone at home, six did not have adolescents, and eight were of current/former FSWs]. Meanwhile, 2.9% (5/171) of households with eligible mother-adolescent pairs declined to participate.

The median age (IQR) of the adolescents was 12 (10–14) years but the adolescents of FSWs were slightly younger than the adolescents of non-FSWs (12 [10–13] vs. 13 [10–15] years). Among the participants 62.9% were females, but with no difference in sex between the groups; half (49.6%) were Catholic; up to 92.5% had only primary or no education; and 17% dropped out of school. More adolescents of FSWs were out of school compared to the adolescents of non-FSWs (23.1% vs. 10.9%). Meanwhile, 5.4% of adolescents were sexually active yet more than three-quarters (78.2%) have never tested for HIV [Table 1].

**Table 1:** Socio-demographic characteristics of adolescents who participated in the CARP study

Characteristic	Adolescents of FSWs N (%)	Adolescents of non-FSWs N (%)	Total N (%)
Age in years median (IQR)	12.0 (10–13)	13.0 (10–15)	12 (10–14)
Sex			
Male	58 (39.5)	51 (34.7)	109 (37.1)
Female	89 (60.5)	96 (65.3)	185 (62.9)
Education level			
≤Primary	138 (93.9)	134 (91.2)	272 (92.5)
Secondary	9 (6.1)	13 (8.8)	22 (7.5)
Currently in school			
No	34 (23.1)	16 (10.9)	50 (17.0)
Yes	113 (76.9)	131 (89.1)	244 (83.0)
Religion			
Catholic	71 (48.3)	75 (51.0)	146 (49.7)
Protestant	12 (8.2)	22 (15.0)	34 (11.5)
Other Christian	50 (34.0)	41 (27.9)	91 (31.0)
Muslim	14 (9.5)	9 (6.1)	23 (7.8)
Sexually active			
No	142 (96.6)	136 (92.5)	278 (94.6)
Yes	5 (3.4)	11 (7.5)	16 (5.4)

### Socio-demographic characteristics of mothers of adolescents in the CARP study

The median age of mothers was 30 (27–35) years, though FSWs were slightly younger than non-FSWs (29 [27–32] vs. 32 [28–37]) years. The majority (89.1%) of mothers were living in rented housing, but more FSWs than non-FSWs (98.6% vs. 81.6%) were in rented housing. Most mothers (51.0%) were divorced, but more FSWs reported being either never married (22.5% vs. 2.0%) or divorced (55.1% vs. 46.9%) than non-FSWs. More FSWs than non-FSWs were main household income earners (92.5% vs. 71.4%). Most mothers (41.5%) were earning below the lowest-earning quartile (≤ Ush100, 000 [US$ 30]), though there was no variation in earnings between FSWs and non-FSWs. More FSWs than non-FSWs reported drinking alcohol (76.2% vs. 12.2%), getting drunk in the previous six months (74.8% vs. 4.8%), and using street drugs (33.6% vs. 1.4%). It is worth noting that compared to non-FSWs, more FSWs left their children to sleep alone in the house without any adult (51.7% vs. 4.8%). Lastly, one-third (33.3%) of mothers were living with HIV, and FSWs were most affected compared to the non-FSWs (40.1% vs. 26.5%*)* [Table 2].

**Table 2:** Socio-demographic characteristics of mothers of adolescents who participated in the CARP study

Characteristic	FSWs N (%)	Non-FSWs N (%)	Total (%)
Median age (IQR) in years	29 (27–32)	32 (28–37)	30 (27–35)
Education level			
≤Primary	102 (69.4)	107 (72.8)	209 (71.1)
Secondary	45 (30.6)	40 (27.2)	85 (28.9)
Live in a rented housing			
No	5 (3.4)	27 (18.4)	32 (10.9)
Yes	142 (96.6)	120 (81.6)	262 (89.1)
Marital status			
Never married	33 (22.5)	3 (2.0)	36 (12.3)
Married/cohabiting	8 (5.4)	47 (32.0)	55 (18.7)
Divorced	81 (55.1)	69 (46.9)	150 (51.0)
Widowed	25 (17.0)	28 (19.1)	53 (18.0)
Highest household income earner			
Mother	136 (92.5)	105 (71.4)	241(82.0)
A household member	11 (7.5)	42 (28.6)	53 (18.0)
Mother’s monthly income (Shillings)			
Q1: ≤100,000	66 (44.9)	56 (38.1)	122 (41.5)
Q2: >100,000-≤150,000	27 (18.4)	32 (21.8)	59 (20.1)
Q3: >150,000-≤210,000	18 (12.2)	23 (15.7)	41 (14.0)
Q4: >2100,000	36 (24.5)	36 (24.5)	72 (24.5)
Have another adult in the household			
No	110 (74.8)	90 (61.2)	200 (68.0)
Yes	37 (25.2)	57 (38.8)	94 (32.0)
Was drinking alcohol during the last six month			
No	35 (23.8)	129 (87.8)	164 (55.8)
Yes	112 (76.2)	18 (12.2)	130 (44.2)
Ever got drunk during the last six months			
No	40 (27.2)	140 (95.2)	180 (61.2)
Yes	107 (72.8)	7 (4.8)	114 (38.8)
Use street drug			
No	97 (66.4)	145 (98.6)	242 (82.6)
Yes	49 (33.6)	2 (1.4)	51 (17.4)
Left children to sleep alone without an adult			
No	13 (8.8)	118 (80.3)	131 (44.6)
Sometimes	58 (39.5)	22 (15.0)	80 (27.2)
Always	76 (51.7)	7 (4.8)	83 (28.2)
HIV status			
Negative	88 (59.9)	108 (73.5)	196 (66.7)
Positive	55 (40.1)	39 (26.5)	98 (33.3)

### Prevalence of childhood victimization among adolescents

Almost all (99.3%) adolescents reported at least one form of lifetime victimization. On average, each adolescent experienced 12.4 of the 34 different types of victimization. The most commonly reported forms of victimization were caregiver victimization (95.2%), conventional crime (94.6%), and witnessed/indirect victimization (92.2%). Sexual victimization (24.5%) was the least reported form of victimization. The adolescents of FSWs suffered from more forms of victimization (median [IQR]) in their lifetime than the adolescents of non-FSWs (13.4[6.4] vs. 11.5[5.1]; *p < 0.01*). Older adolescents experienced more victimization than their younger counterparts (14.0([5.4] vs. 11.7[5.9]; p < 0.01). Likewise, male adolescents reported more victimization (mean [IQR]) than their female counterparts (13.4[5.8] vs. 11.9[5.8]; p < 0.05).

#### Caregiver victimization

Caregiver victimization was the most reported type of victimization, with 95.2% of adolescents reporting it. The most and least commonly reported forms of caregiver victimization were physical abuse (91.5%) and custodian interference (21.8%). The adolescents of non-FSWs experienced more caregiver victimization than the adolescent children of FSWs (98.0% vs. 92.5%; p < 0.05). Furthermore, more adolescents of non-FSWs than adolescents of FSWs were physically abused (96.6% vs. 86.4%; *p < 0.01*). Conversely, more adolescents of FSWs were emotionally abused (65.8% vs. 50.0%; *p < 0.05*) and neglected (37.4% vs. 21.1%; *p < 0.01*) than adolescents of non-FSWs. Overall, caregiver victimization did not show significant variations with age and sex, but older adolescents reported more physical (96.7% vs. 89.1%; p < 0.05) and emotional abuse (68.5% vs. 53.7%) than their younger counterparts.

#### Conventional crime

Conventional crime was the second most common form of victimization, with 94.6% of participants reporting it. The most common forms of conventional crime were personal theft (84.0%) and vandalism (72.4%). While the least common conventional crimes were kidnap (10.2%) and bias attacks (1.7%). There were no significant differences in conventional crimes between the two adolescents of FSWs and the comparison groups. However, the adolescents of FSWs reported more cases of kidnap than their comparators (15.8% vs. 4.8%; *p < 0.01*). Further analysis revealed that older adolescents reported more personal theft (92.4% vs. 80.2%; p < 0.01), vandalism (83.5% vs. 67.3%; p < 0.01), and assault without a weapon (79.4% vs. 58.9; P < 0.01). Meanwhile, male adolescents reported more robbery (73.4% vs. 59.5%; p < 0.05) and personal theft (90.8% vs. 80%; p < 0.05) than their female counterparts.

#### Witnessed/indirect victimization

92.2% of the participants experienced witnessed/indirect victimization. The commonest forms of witnessed/indirect victimization were witnessing assault without a weapon (78.9%) and witness to assault with a weapon (68.0%). The least generic form of witnessed/indirect victimization was witness to random shooting (5.4%). Compared to the adolescents of non-FSWs, adolescents of FSWs reported more exposures to murder scenes (42.9% vs. 26.5%; *p < 0.01*), witness to domestic violence (39.5% vs. 26.5%; *p < 0.05*), and witness to the murder of a relative (31.3% vs. 21.1%; *p < 0.05*). Overall, older adolescents reported more witnessed/indirect victimization than younger adolescents (98.9% vs. 89.1%; p < 0.01). Specifically, older adolescents reported witness to domestic violence (41.3% vs. 29.2%; p < 0.05) and witness to assault without a weapon (89.1% vs. 74.3%; p < 0.01) than the younger adolescents. Nevertheless, exposure to witness/indirect victimization did not vary by sex.

#### Peer and sibling victimization

Slightly more than three-quarters (77.9%) of adolescents reported peer/sibling victimization, with no significant variation between the two study groups. The most reported peer/sibling victimizations were verbal aggression (57.8%) and peer/sibling assault (46.7%). Meanwhile, the least reported peer/sibling victimization was physical intimidation (7.1%) and dating violence (7.1%). Specifically, adolescents of FSWs reported more physical intimidation (10.2% vs. 4.1%; *p < 0.05*), relational aggression (36.4% vs. 18.4%; *p < 0.01*), and verbal aggression (68.7% vs. 46.9%; *p < 0.001*) than adolescent of non-FSWs. Male adolescents reported more peer/sibling victimization than their female counterparts (84.4% vs. 74.1%; p < 0.05). Furthermore, older adolescents reported more peer/sibling assault (57.6% vs. 44.6%; p < 0.05), verbal aggression (68.5% vs. 53.0%; p < 0.05) and dating violence (12.0% vs. 5.0%; p < 0.05%).

#### Sexual victimization

Sexual victimization was the least reported form of victimization, with 24.5% of adolescents experiencing it. Adolescents of FSWs were more likely to experience sexual victimization than adolescent children of non-FSWs (31.3% vs. 17.7%; *p < 0.01*). The most common sexual victimization was verbal sexual harassment (12.9%). Adolescents of FSWs reported more verbal sexual harassment than adolescents of non-FSWs (20.4% vs. 5.4%: p < 0.001) [Table 3]. More sexual victimization was reported among males (31.2% vs. 20.5%; p < 0.05) and older (34.8% vs. 19.8%; p < 0.01) adolescents. Specifically, older adolescents reported more sexual assault by a known adult (9.8% vs. 3.5%; p < 0.05), sexual assault by peer/sibling (12.0% vs. 5.4%; p < 0.01), and statutory rape (8.7% vs. 2.0%; p < 0.01). Likewise, more male adolescents reported verbal sexual harassment (22.9% vs. 7.0%; p < 0.001) and sexual assault by peers/siblings (10.1% vs. 2.7%; p < 0.01) than their female counterparts.

**Table 3:** Juvenile victimization among adolescents in post-conflict Gulu City

Characteristic	Is the mother a sex worker?				Adolescent’s age (years)				Adolescent’s sex				
	Yes N (%)	No N (%)	PPD (%)	p-value	10–13 N (%)	14–17 N (%)	PPD (%)	p-value	Male N (%)	Female N (%)	PPD (%)	P-value	
***C. Conventional crime***	140(95.2)	138(93.9)	1.3	0.607	188(93.1)	90(97.8)	-4.7	0.095	105(96.3)	173(93.5)	2.8	0.304	
C1.Robbery	97(66.0)	93(63.3)	2.7	0.626	125(61.9)	65(70.7)	-8.8	0.145	80(73.4)	110(59.5)	13.9	0.016	
C2.Personal theft	127(86.4)	120(81.6)	4.8	0.265	162(80.2)	85(92.4)	-12.2	0.008	99(90.8)	148(80.0)	10.8	0.014	
C3.Vandalism	103(70.6)	109(74.1)	-3.5	0.491	136(67.3)	76(83.5)	-16.2	0.004	86(78.9)	126(68.5)	10.4	0.054	
C4.Assault with weapon	87(59.2)	79(53.7)	5.5	0.347	114(56.4)	52(56.5)	-0.1	0.989	63(57.8)	103(55.7)	2.1	0.723	
C5. Assault without weapon	97(66.0)	95(64.6)	1.4	0.806	119(58.9)	73(79.4)	-20.5	0.001	77(70.6)	115(62.2)	8.4	0.140	
C6.Attempted assault	91(61.9)	100(68.0)	-6.1	0.271	127(62.9)	64(69.6)	-6.7	0.265	78(71.6)	113(61.1)	10.5	0.069	
C7.Kidnap	23(15.8)	7(4.8)	11.0	0.002	24(11.9)	6(6.5)	5.4	0.156	13(11.9)	17(9.2)	2.7	0.463	
C8.Bias attack	5(3.4)	0(0)	3.4	0.060	3(1.5)	2(2.2)	-0.7	0.672	3(2.8)	2(1.1)	1.7	0.284	
***M. Caregiver victimization***	136(92.5)	144(98.0)	-5.5	0.028	190(94.1)	90(97.8)	-3.7	0.160	107(98.2)	173(93.5)	4.7	0.070	
M1.Physical abuse	127(86.4)	142(96.6)	-10.2	0.002	180(89.1)	89(96.7)	-7.6	0.030	103(94.5)	166(89.7)	4.8	0.157	
M2.Emotional abuse	96(65.8)	75(50.0)	15.8	0.011	108(53.7)	63(68.5)	-14.8	0.017	68(63.0)	103(55.7)	7.3	0.222	
M3.Physical/emotional neglect	55(37.4)	31(21.1)	16.3	0.002	57(28.2)	29(31.5)	-3.3	0.564	38(34.9)	48(26.0)	8.9	0.105	
M4.Custodian interference	38(25.8)	26(17.7)	8.1	0.090	42(20.8)	22(23.9)	-3.1	0.548	19(17.4)	45(24.3)	-6.9	0.167	
***P. Peer/sibling victimization***	120(81.6)	109(74.1)	7.5	0.122	151(74.8)	78(84.8)	-10.0	0.055	92(84.4)	137(74.1)	10.3	0.039	
P1.Gang/group assault	56(38.1)	52(35.4)	2.7	0.628	71(35.1)	37(40.2)	-5.1	0.403	46(42.2)	62(33.5)	8.7	0.136	
P2.Peer/sibling assault	78(53.0)	65(44.2)	8.8	0.129	90(44.6)	53(57.6)	-13.0	0.038	54(49.5)	89(48.1)	1.4	0.812	
P3.Physical intimidation	15(10.2)	6(4.1)	6.1	0.042	12(5.9)	9(9.8)	-3.9	0.236	9(8.3)	12(6.5)	1.8	0.569	
P4.Relational aggression	53(36.0)	27(18.4)	17.6	0.001	57(28.2)	23(25.0)	3.2	0.565	34(31.2)	46(24.9)	6.3	0.239	
P5.Verbal aggression	101(68.7)	69(46.9)	21.8	< 0.001	107(53.0)	63(68.5)	-15.5	0.013	71(65.1)	99(53.5)	11.6	0.051	
P6.Dating violence	21(7.1)	21(7.1)	0	0.258	10(5.0)	11(12.0)	-7.0	0.031	10(9.2)	11(6.0)	3.2	0.299	
*** S. Sexual victimization***	46(31.3)	26(17.7)	13.6	0.007	40(19.8)	32(34.8)	-15.0	0.006	34(31.2)	38(20.5)	10.7	0.040	
S1.Sexual assault by a known adult	11(7.5)	5(3.4)	4.1	0.123	7(3.5)	9(9.8)	-6.3	0.027	6(5.5)	10(5.4)	0.1	0.971	
S2.Sexual assault by an unknown adult	8(5.4)	2(1.4)	4.0	0.103	5(2.5)	5(5.4)	-2.9	0.194	3(2.8)	7(3.8)	-1.0	0.637	
S3.Sexual assault by peer/sibling	8(5.4)	8(5.4)	0	1.000	5(2.5)	11(12.0)	-9.5	0.001	11(10.1)	5(2.7)	7.4	0.007	
S4.Forced sex includes an attempt	14(9.5)	6(4.1)	5.4	0.064	11(5.5)	9(9.8)	-4.3	0.171	5(4.6)	15(8.1)	-3.5	0.247	
S5.Flashing/sexual exposure	16(10.8)	10(6.8)	4.0	0.218	16(7.9)	10(10.9)	-3.0	0.409	11(10.1)	15(8.1)	2.0	0.563	
S6.Verbal sexual harassment	30(20.4)	8 (5.4)	15.0	< 0.001	24(11.9)	14(15.2)	-3.3	0.429	25(22.9)	13(7.0)	15.9	< 0.001	
S7.Statutory rape	4(2.7)	8(5.4)	-2.7	0.238	4(2.0)	8(8.7)	-5.7	0.007	4(3.7)	8(4.3)	-0.6	0.784	
*** W. Witnessed/indirect victimization***	137(93.2)	134(91.2)	2.0	0.515	180(89.1)	91(98.9)	-9.8	0.004	102(93.6)	169(91.3)	2.3	0.492	
W1.Witness to domestic violence	58(39.5)	39(26.5)	13.0	0.018	59(29.2)	38(41.3)	-12.1	0.041	39(35.8)	58(31.3)	4.5	0.435	
W2.Witness to parent assaulting	80(54.4)	73(49.7)	4.7	0.414	99(49.0)	54(58.7)	-9.7	0.123	56(51.4)	97(52.4)	1.0	0.861	
W3.Witness to assault with a weapon	96(65.3)	104(70.8)	-5.5	0.317	131(64.9)	69(75.0)	-10.1	0.084	79(72.5)	121(65.4)	7.1	0.209	
W4.Witness to assault without a weapon	115(78.2)	117(79.6)	-1.4	0.775	150(74.3)	82(89.1)	-14.8	0.004	88(80.7)	144(77.8)	2.9	0.557	
W5.Burglary of family household	97(66.0)	83(56.5)	-9.5	0.094	125(61.9)	55(59.8)	2.1	0.732	73(67.0)	107(57.8)	9.2	0.121	
W6.Witness to the murder of a relative	46(31.3)	31(21.1)	10.2	0.047	48(23.8)	29(31.5)	-7.7	0.161	35(32.1)	42(22.7)	9.4	0.076	
W7.Exposure to a murder scene	63(42.9)	39(26.5)	16.4	0.003	69(34.2)	33(35.9)	-1.7	0.775	40(36.7)	62(33.5)	3.2	0.580	
W8.Exposure to war	50(34.0)	47(32.0)	2.0	0.710	61(30.2)	36(39.1)	-8.9	0.131	29(26.6)	68(36.8)	-10.2	0.074	
W9.Witness to random shooting	10(6.8)	5(3.4)	3.4	0.185	8(4.0)	7(7.6)	-3.6	0.187	6(5.5)	9(4.9)	-0.6	0.810	
***Mean victimization score*** (mean [SD])	13.4(6.4)	11.5(5.1)	1.9	0.005	11.7(5.9)	14.0(5.4)	-2.3	0.002	13.4(5.8)	11.9(5.8)	1.5	0.030	

PPD = Percentage point difference; Mean victimization score was measured out of 34

## Discussion

Understanding the epidemiology of victimization among CARP is of paramount importance since untreated childhood victimization can lead to devastating short-term and often long-lasting negative impacts on survivors’ physical and mental health. To the best of our knowledge, there is only limited information on childhood victimization among children of FSWs. Thus, our study is the first to comprehensively investigate victimization among adolescents of FSWs and CARP as a whole using an approach by Finkelhor, Ormrod et al. (2004) that enables cross-cultural comparisons [4].

Childhood victimization was found to be highly prevalent (99.3%) among adolescents in Northern Uganda. The current finding reports a much higher prevalence of lifetime victimization than in China [71%] (46), the United States [80%] [47), Spain [83%][48], Mexico [85.5%] [49], and Chile [92.6%] [50]. Partly, this could be attributed to the fact that most parents/guardians in Northern Uganda like to use nonviolent discipline methods, psychological aggression, or corporal punishment to discipline their children [51]. If left unaddressed, this extreme level of victimization can lead to psychopathology and psychological distress during childhood and adult life [52]. This calls for multi-level and family-focused interventions that promptly detect, secure and rehabilitate vulnerable children suffering from victimization. Such interventions can include parenting programs, trauma counselling and other appropriate psychotherapies to break the vicious cycle of victimization, psychological distress and re-victimization [53]. Likewise, the government and development partners need to develop preventive mechanisms targeting the rampant victimization among adolescents through education programs, child-centred parenting, community support programs, and linking affected individuals with existing child protection services [54].

On average, each adolescent experienced 12.4 out of 34 possible types of victimization as measured by JVQ. This is far above the average (3.7%) lifetime victimization reported in the United States [55]. This is partly explained by the fact that Ugandan adolescents are rampantly exposed to nonviolent discipline methods, psychological aggression, and corporal punishment by parents, teachers and other members of society [51] despite the government ban [56]. Unsparingly, the current study showed that the most reported forms of victimization by adolescents were: caregiver victimization, conventional crime, and witnessed/indirect victimization. Secondly, the two decades of armed conflict (1986–2006) between the Lord’s Resistance Army (LRA) and the Ugandan government could have negatively impacted adolescents’ exposure to victimization [51] by fostering beliefs and traditions that support corporal punishment to discipline children [57]. Hence, the beliefs, traditions and practices that perpetuate corporal punishment against children need to be strongly discouraged through targeted education, dialogue, and implementation of the national law against corporal punishment.

This was the first study to extensively examine lifetime victimization among adolescents of FSWs. Within all the five domains of JVQ, the adolescents of FSWs experienced higher rates of and more severe victimization than adolescents of non-FSWs. We postulate that this disproportionate level of victimization among adolescents of FSWs could be perpetuated by the pervasive nature of sex work stigma, the toxic legal environment in which FSWs operate, and the poor mental health of FSWs [58]. The high levels of lifetime victimization among adolescents of FSWs are concerning and need urgent remedy. If left untreated many of these victims will grow into adults who commit crimes including sex offences [59, 60] and suffer from mental illnesses like depression [61].

Lastly, findings revealed that male and older adolescents were more likely to report lifetime victimization than their female and young counterparts. Several studies also showed that older adolescents experience more victimization than younger ones [47–49]. This is because victimization accumulates with age [55] as children start to play outdoors as well as go to school. In addition, older children may not get much attention and protection from parents, older siblings and the community leaving them exposed to a risky environment. Although some studies showed no sex variation in exposure to victimization [48, 62], many are in agreement with the current findings showing that male adolescents were at greater risk of victimization than female adolescents [46, 63, 64]. In Northern Uganda, male adolescents are expected to defend themselves since they are expected to be defenders of their families. In Mexico, boys were involved in more outdoor activities which tend to be riskier, thus getting exposed to peer violence, conventional crime and witness victimization [65].

### Strengths and limitations of the study

This study was cross-sectional, thus precluding inferring causality. Second, the adolescents of FSWs were recruited through their mothers with help of respondent-driven sampling, thus they might not be a true representative of the general population of adolescents of FSWs in the region. Nonetheless, we ensured that the seeds come from diverse representative communities of FSWs to improve on generalizability. The data may have been negatively influenced by recall bias since we asked about personal experiences of traumatizing events that might have been difficult to answer. Nevertheless, through the robust involvement of FSWs throughout the study conduct, we developed trust that reduced such information bias. Lastly, this study might have been affected by some residual confounders not captured in this study.

## Conclusions

There is a considerable knowledge gap in the healthcare needs of children and adolescents of FSWs. Thus, we set out to determine the impact of maternal sex work on adolescent victimization in Northern Uganda. Victimization is highly prevalent among adolescents in Northern Uganda and disproportionately affects the adolescents of FSWs. Government and development partners need to urgently develop policies and interventions targeting prevention, early detection, and timely management of victimization among the children and adolescents of FSWs. Lastly, there is also a need for longitudinal studies to understand the long-term impacts of childhood victimization.

## Electronic supplementary material

Below is the link to the electronic supplementary material.

Supplementary Material 1

## Data Availability

All data generated/analyzed are included in this published article [and its supplementary information file].

## List of Abbreviations

CRAP: Children of At-Risk Parents
HIV: Human Immunodeficiency Virus
IQR: Interquartile Range
JVQ: Juvenile Victimization Questionnaire
SD: Standard Deviation
FSWs: Female Sex Workers
TASO: The AIDS Support Organization
